# Change in Food Intake Frequency at Five Years after Baseline in the JACC Study

**DOI:** 10.2188/jea.15.S48

**Published:** 2005-05-16

**Authors:** Sadao Suzuki, Miyuki Kawado, Shuji Hashimoto, Shinkan Tokudome, Takesumi Yoshimura, Akiko Tamakoshi

**Affiliations:** 1 Department of Health Promotion and Preventive Medicine, Nagoya City University Graduate School of Medical Sciences.; 2 Department of Hygiene, Fujita Health University School of Medicine.; 3 Fukuoka Institute of Health and Environmental Sciences.; 4 Department of Preventive Medicine/Biostatistics and Medical Decision Making, Nagoya University Graduate School of Medicine.

**Keywords:** epidemiologic method, food, questionnaire, cohort study, Japan

## Abstract

BACKGROUND: In a cohort study, information on an individual is taken at baseline, after which it usually remains fixed. There is some risk that this will lead to misclassification and cause weakened or biased results. To prevent such distortion, following up of exposure is important, although it is still scarce in practice.

METHODS: In the Japan Collaborative Cohort Study for Evaluation of Cancer Risk (JACC Study) sponsored by Monbusho (Ministry of Education, Science, Sports and Culture of Japan), 37,838 (14,531 males and 23,307 females) subjects out of a cohort of 127,477 inhabitants answered an interim questionnaire on food intake frequency consisting of 33 items about five years after registration. The long-term reproducibility was assessed using Spearman’s correlation coefficients and agreement. From data at two time points, longitudinal change, age effect, and secular trend were examined. Subjective changes in these items at the time of the interim survey were also compared to longitudinal changes.

RESULTS: Spearman’s correlation coefficients varied from 0.27 (fruit juice in males) to 0.55 (beef in females and milk in males), and agreement from 29.9% (fruit juice in males) to 61.4% (liver in females). Correlation was relatively stronger in meat and dairy products and weaker in vegetables and fruits. In both males and females, most increased food item was edible wild plants followed by confectioneries (males) and yogurt (females).

CONCLUSION: Over five years, food intake was considerably changed. These interim data could be used for a long-term follow-up study to prevent the results becoming weakened or biased.

For a self-administered food frequency questionnaire, short-term reproducibility needs to be validated to prevent misclassification of true food intake.^1^ However, long-term reproducibility is decreased not only by low reproducibility in the short term but also by real intake changes over time.^2^ In a cohort study, information on an individual is taken at baseline and then usually remains fixed. However, if exposure changes over time, misclassification occurs which might cause weakened or biased results.^3^^,^^4^ To prevent such distortion, following up of food intake over the long term is important, although in practice this is still scarce.^5^^-^^7^ In the Japan Collaborative Cohort Study for Evaluation of Cancer Risk (JACC Study) sponsored by Monbusho (Ministry of Education, Science, Sports and Culture of Japan), an interim survey was designed to examine the changes in lifestyles. In this paper, the authors discuss long-term reproducibility and change in intake frequency of 33 food items over five years.

## METHODS

JACC Study is a large-scale multi-center cohort study, which aims to clarify the etiology of cancer mortality and incidence. Baseline information on physical status and lifestyle, as well as medical history, family history, education, and occupation, was gathered between 1988 and 1990 using a self-administered questionnaire. Baseline data are for 127,477 inhabitants (54,032 males and 73,445 females) enrolled from 45 study areas throughout Japan.^9^ About 5 years after the baseline survey, interim survey about lifestyle factors was conducted. Interim survey was asked to every participant in 18 areas. In contrast, it was asked to some of the cohort subjects in 13 areas, where for example, only examinees of basic health examinations approximately five years after the baseline survey, conducted under the Health and Medical Service Law for the Aged, were invited to the interim survey.^10^ In 14 areas interim survey was not conducted. The research was also done by using a self-administered questionnaire, including demographic information, past medical history, family cancer history in these 5 years, exercise/sports activities, frequency of food intake and change of intake compared with 5 years before, smoking and alcohol drinking status and so on. Out of 110,792 subjects between 40-79 years old at the time of registration, 46,680 (42.1%) individuals answered the interim questionnaire. Table 1 shows the number and response rate by how the interim survey was conducted. For 18 areas in which the interim questionnaire was asked to every participant, the response rate was 78.8%, while for the 13 area, in which it was not asked to all participants, the response rate was only 24.1%.

**Table 1.** The number of the participants of the baseline and interim survey.

Target of interim survey	Baseline survey	Interim survey	
All participants of the baseline survey (18 areas)	48,016	37,853	(78.8%)
Some participants of the baseline survey (13 areas)	36,460	8,797	(24.1%)
No participants (interim survey was not conducted) (14 areas)	26,316	0	(0.0%)

Total (45 areas)	110,792	46,650	(42.1%)

Among them, 37,838 (14,531 males and 23,307 females) were eligible subjects who answered an interim questionnaire on food intake frequency. In some areas, several items were not included in the questionnaire, and those areas were excluded from the analysis by food items.

In the baseline and interim surveys, the subjects were asked average intake frequency of the same 33 food items in a past year. They chose one appropriate frequency among five categories, i.e., (1) almost none, (2) 1-2 times per month, (3) 1-2 times per week, (4) 3-4 times per week, and (5) almost every day. Scores one to five were used to evaluate the individual’s food intake frequency, and the long-term reproducibility was assessed using Spearman’s correlation coefficients and agreement (exact agreement and agreement allowing one category difference). Longitudinal change in intake frequency of food items was measured by the difference in scores on the two questionnaires.

In order to observe the difference in the change in food intake frequency by age, we divided the subjects into eight age specific sub-cohorts (40-44, 45-49, 50-54, 55-59, 60-64, 65-69, 70-74, and 75-79 years old). The variation of two scores for food intake frequency over five years consists of two parts: age effect and secular trend. We assumed the difference from a sub-cohort to one rank older at baseline as the age effect for five years, and that secular trend could be calculated as the longitudinal variation subtracted by the age effect. From this analysis, subjects aged 75-79 years old were excluded since there was no older age group at baseline. Each analysis was performed by sex.

After the long-term reproducibility and variation assessment of 33 food items using Spearman’s correlation coefficients, agreement of the answer, longitudinal difference, age effect, and secular trend, we checked whether the results were consistent between males and females using Spearman’s correlation coefficients of males and females for the indexes mentioned above.

In the interim questionnaire, subjective changes in intake were also asked for the same 33 items. The scores were 1 for ‘increased’, 0 for ‘not changed’, and -1 for ‘decreased’. We examined the consistency of the food frequency variations taken from two different methods, i.e., the difference on two questionnaires and subjective changes at the time of the interim questionnaire using Spearman’s correlation coefficients by sex. All analyses were performed using SAS^®^ version 8.2 (SAS Institute).

Our entire study design, which comprised singular and collective use of epidemiologic data and biological materials (serum only), was approved in 2000 by the Ethical Board at Nagoya University School of Medicine, where the central secretariat of the JACC study is located.

## RESULTS

Table 2 shows the distribution of age and sex of the subjects. The mean age (standard deviation) of males and females was 58.1 (9.6) and 58.0 (9.5) years old, respectively. The mean (standard deviation) period was 4.71 (0.69) years and median was 4.83 years.

**Table 2.** Age and sex distribution of the subjects.

Age (year)	Males		Females		Total	
40-44	1,569	(10.8%)	2,332	(10.0%)	3,901	(10.3%)
45-49	1,545	(10.6%)	2,690	(11.5%)	4,235	(11.2%)
50-54	1,854	(12.8%)	3,276	(14.1%)	5,130	(13.6%)
55-59	2,689	(18.5%)	4,183	(17.9%)	6,872	(18.2%)
60-64	3,164	(21.8%)	4,710	(20.2%)	7,874	(20.8%)
65-69	1,827	(12.6%)	3,324	(14.3%)	5,151	(13.6%)
70-74	1,248	(8.6%)	1,795	(7.7%)	3,043	(8.0%)
75-79	635	(4.4%)	997	(4.3%)	1,632	(4.3%)

Total	14,531		23,307		37,838	

Table 3 shows the proportion of food intake frequency at the baseline and interim surveys. Missing values were common, more than 15%, for margarine, yogurt, butter, and cheese intake in both surveys. In contrast, missing values were fairly few, around 5%, for eggs, fresh fish, and tofu intake. Among the 33 items, the proportion of missing values was very consistent not only between males and females at baseline (Spearman’s correlation coefficients: 0.98) and at the interim survey (0.98), but also between baseline and interim questionnaire in both males (0.89) and females (0.89). The occurrence of missing values strongly depended on the items regardless of sex or time.

**Table 3.** Distribution of food intake frequency at baseline and interim survey.

		Males								Females							
	
		Almost none (%)	1-2/month (%)	1-2/week (%)	3-4/week (%)	Almost every day (%)	Subtotal No.	Missing value (%)	Total No.	Almost none (%)	1-2/month (%)	1-2/week (%)	3-4/week (%)	Almost every day (%)	Subtotal No.	Missing value (%)	Total No.
Beef	Baseline	22.7	37.8	30.4	7.8	1.3	12,809	10.8	14,358	26.3	32.2	31.2	9.1	1.1	20,360	11.6	23,040
	Interim	14.9	44.9	33.3	6.2	0.8	13,166	8.3	14,358	20.2	38.6	34.1	6.4	0.8	20,897	9.3	23,040
Pork	Baseline	10.4	25.5	44.0	17.0	3.2	13,222	9.0	14,531	13.3	21.6	44.5	17.3	3.3	20,776	10.9	23,307
	Interim	7.6	31.5	46.5	13.0	1.5	13,096	9.9	14,531	10.2	26.7	47.4	14.3	1.5	20,690	11.2	23,307
Ham and sausages	Baseline	23.1	28.8	33.2	11.4	3.5	12,467	14.2	14,531	26.1	26.8	33.0	11.2	2.9	19,727	15.4	23,307
	Interim	19.6	36.8	32.3	9.2	2.2	12,576	13.5	14,531	21.8	35.6	32.5	8.2	1.9	19,785	15.1	23,307
Chicken	Baseline	9.4	30.1	44.1	14.2	2.1	13,039	10.3	14,531	9.1	24.2	47.4	17.0	2.3	20,862	10.5	23,307
	Interim	8.0	34.0	45.1	11.9	1.0	13,245	8.9	14,531	8.1	28.8	47.8	14.1	1.2	21,115	9.4	23,307
Liver	Baseline	41.4	44.2	10.3	3.4	0.6	11,739	14.7	13,756	48.6	38.8	8.7	3.1	0.7	18,787	14.9	22,084
	Interim	39.5	48.1	9.8	1.9	0.6	11,810	14.1	13,756	47.4	41.6	8.8	1.6	0.5	18,691	15.4	22,084
Eggs	Baseline	2.1	5.5	23.2	24.5	44.7	13,913	4.3	14,531	2.5	5.0	24.7	26.5	41.3	22,233	4.6	23,307
	Interim	1.6	5.7	23.7	30.8	38.2	13,716	5.6	14,531	2.1	6.3	25.9	32.6	33.1	21,885	6.1	23,307
Milk	Baseline	20.4	9.6	14.7	13.0	42.3	12,813	8.6	14,024	17.8	7.2	13.5	13.1	48.3	20,689	7.8	22,443
	Interim	17.6	10.2	13.3	13.4	45.6	12,792	8.8	14,024	14.2	6.6	11.5	14.4	53.3	20,608	8.2	22,443
Yogurt	Baseline	70.4	14.3	7.6	3.5	4.2	11,383	18.8	14,024	54.0	19.8	14.2	6.1	5.9	18,471	17.7	22,443
	Interim	59.0	19.8	10.8	4.6	5.9	11,847	15.5	14,024	37.7	23.8	18.9	10.0	9.6	19,216	14.4	22,443
Cheese	Baseline	54.9	27.3	11.4	3.9	2.5	11,591	16.8	13,929	57.1	24.3	11.9	4.2	2.5	18,570	16.9	22,351
	Interim	46.7	33.9	12.8	3.8	2.8	11,785	15.4	13,929	47.5	30.6	14.1	4.7	3.2	18,769	16.0	22,351
Butter	Baseline	55.7	23.1	12.6	4.5	4.1	11,511	17.4	13,929	53.8	21.6	14.3	5.4	5.0	18,360	17.9	22,351
	Interim	50.9	30.1	12.8	3.4	2.7	11,684	16.1	13,929	49.6	28.8	14.1	4.3	3.2	18,528	17.1	22,351
Margarine	Baseline	47.6	20.9	15.6	6.7	9.2	11,279	19.0	13,929	36.3	19.1	20.0	9.7	14.9	18,193	18.6	22,351
	Interim	47.4	24.7	13.9	5.9	8.1	11,665	16.3	13,929	36.5	23.4	18.9	9.2	11.9	18,840	15.7	22,351
Deep fried foods	Baseline	4.1	25.7	46.9	18.4	4.9	12,659	12.9	14,531	4.0	27.6	46.8	17.4	4.2	20,324	12.8	23,307
	Interim	2.9	25.1	49.7	19.0	3.3	13,381	7.9	14,531	3.2	27.8	49.1	17.3	2.5	21,423	8.1	23,307
Fried vegetables	Baseline	3.5	17.6	41.7	24.1	13.1	12,734	12.4	14,531	3.6	16.1	41.3	25.2	13.9	20,637	11.5	23,307
	Interim	2.3	15.3	41.1	30.2	11.1	13,539	6.8	14,531	2.3	14.2	40.3	30.8	12.3	21,764	6.6	23,307
Flesh fish	Baseline	1.4	7.6	33.8	30.3	26.9	13,903	4.3	14,531	1.7	6.6	32.4	32.5	26.9	22,146	5.0	23,307
	Interim	1.0	7.7	33.2	36.0	22.1	13,765	5.3	14,531	1.5	7.1	30.8	39.3	21.4	21,930	5.9	23,307
Dried/salted fish	Baseline	8.3	25.3	37.9	18.5	9.9	13,236	8.9	14,531	10.7	26.0	35.6	18.0	9.7	20,794	10.8	23,307
	Interim	4.9	23.4	40.5	22.0	9.3	13,398	7.8	14,531	6.2	24.4	39.3	21.7	8.5	21,360	8.4	23,307
Boiled fish paste	Baseline	23.4	34.0	29.0	10.3	3.2	11,886	13.1	13,685	20.1	33.3	31.9	11.6	3.1	19,048	13.2	21,947
	Interim	19.7	41.5	28.8	8.3	1.7	12,119	11.4	13,685	16.9	41.5	30.9	9.0	1.7	19,446	11.4	21,947
Green-leafy vegetables	Baseline	1.4	8.5	29.9	28.1	32.1	12,947	7.1	13,929	0.9	5.5	26.2	29.9	37.5	20,696	7.4	22,351
	Interim	1.0	8.4	30.4	34.9	25.4	13,161	5.5	13,929	0.6	5.5	26.2	36.7	30.9	21,129	5.5	22,351
Carrots and squash	Baseline	5.2	20.9	37.7	23.9	12.4	13,347	8.1	14,531	1.4	11.4	34.8	32.0	20.4	21,637	7.2	23,307
	Interim	2.4	19.2	39.4	27.8	11.3	13,417	7.7	14,531	0.8	9.5	34.4	36.7	18.6	21,578	7.4	23,307
Tomatoes	Baseline	13.2	26.1	30.3	18.3	12.1	13,095	9.9	14,531	11.5	21.3	30.0	20.2	17.0	20,824	10.7	23,307
	Interim	8.6	25.0	33.1	21.6	11.7	13,041	10.3	14,531	6.8	19.1	30.9	25.3	17.9	20,763	10.9	23,307
Cabbage and lettuce	Baseline	1.9	10.3	34.4	30.5	22.8	13,600	6.4	14,531	1.4	6.6	29.4	31.8	31.0	21,903	6.0	23,307
	Interim	1.4	9.8	36.1	35.7	16.9	13,584	6.5	14,531	1.1	6.9	30.2	38.7	23.1	21,868	6.2	23,307
Chinese cabbage	Baseline	4.4	17.6	37.6	25.5	14.8	11,720	14.4	13,685	5.9	16.8	35.7	24.8	16.9	18,402	16.2	21,947
	Interim	4.5	20.4	38.2	25.4	11.5	12,401	9.4	13,685	5.6	22.3	36.6	23.7	11.7	19,551	10.9	21,947
Wild plants	Baseline	37.2	40.0	14.1	6.4	2.3	12,291	11.8	13,929	42.5	35.7	12.9	6.5	2.4	19,290	13.7	22,351
	Interim	20.2	42.0	24.3	9.8	3.8	12,301	11.7	13,929	20.8	41.6	23.6	9.9	4.1	19,442	13.0	22,351
Mushrooms	Baseline	6.8	35.5	35.6	15.9	6.1	11,959	12.6	13,685	4.3	25.7	38.9	22.6	8.5	19,160	12.7	21,947
	Interim	5.8	35.1	38.2	15.9	5.0	12,369	9.6	13,685	4.4	25.9	39.5	22.3	7.9	19,858	9.5	21,947
Potatoes	Baseline	5.1	24.4	38.9	20.9	10.7	13,443	7.5	14,531	1.4	13.5	39.2	29.0	16.9	21,783	6.5	23,307
	Interim	3.1	23.1	39.3	24.6	9.8	13,373	8.0	14,531	1.3	14.1	39.0	31.9	13.8	21,514	7.7	23,307
Seaweeds	Baseline	1.7	13.7	32.8	27.2	24.6	13,635	6.2	14,531	1.1	7.8	27.6	29.0	34.5	21,855	6.2	23,307
	Interim	1.9	16.7	35.6	28.6	17.3	13,417	7.7	14,531	1.3	10.4	31.3	33.7	23.3	21,489	7.8	23,307
Pickled vegetables	Baseline	5.8	6.6	14.3	16.5	56.8	13,555	6.7	14,531	5.9	5.8	12.4	14.9	61.0	21,728	6.8	23,307
	Interim	4.5	6.3	13.5	18.0	57.8	13,521	7.0	14,531	4.2	5.7	11.5	16.5	62.2	21,510	7.7	23,307
Tsukudani (food boiled with soy)	Baseline	24.2	29.6	27.4	12.1	6.6	12,778	12.1	14,531	28.8	28.6	24.2	11.9	6.6	20,294	12.9	23,307
	Interim	15.9	34.4	30.4	13.4	5.9	12,911	11.1	14,531	18.7	33.7	28.1	13.2	6.2	20,522	11.9	23,307
Boiled beans	Baseline	22.7	38.2	24.2	10.6	4.2	11,623	15.1	13,685	15.7	42.0	24.6	12.2	5.5	18,804	14.3	21,947
	Interim	14.6	43.1	27.4	10.6	4.3	12,176	11.0	13,685	9.8	44.7	28.3	12.3	4.9	19,574	10.8	21,947
Tofu (soybean curd)	Baseline	1.3	6.8	32.9	32.2	26.9	13,905	4.3	14,531	1.0	4.8	28.9	32.6	32.7	22,272	4.4	23,307
	Interim	0.9	5.8	29.0	37.3	27.0	13,761	5.3	14,531	0.8	4.6	24.6	38.9	31.1	22,037	5.4	23,307
Oranges	Baseline	8.5	18.5	27.4	22.1	23.4	13,333	8.2	14,531	4.2	10.0	21.5	22.5	41.8	21,573	7.4	23,307
	Interim	6.5	22.2	29.5	22.9	18.9	13,263	8.7	14,531	3.6	14.0	25.3	26.0	31.1	21,393	8.2	23,307
Fruits other than oranges	Baseline	5.5	15.5	29.5	24.2	25.3	12,766	12.1	14,531	3.4	8.2	20.4	25.1	42.9	20,325	12.8	23,307
	Interim	4.3	18.2	30.4	25.6	21.5	13,131	9.6	14,531	2.3	9.8	23.4	29.3	35.1	21,085	9.5	23,307
Fruit juice	Baseline	20.5	18.5	26.9	19.0	15.1	12,234	15.8	14,531	22.5	16.2	23.1	18.1	20.2	19,317	17.1	23,307
	Interim	27.6	30.8	24.6	11.3	5.7	12,800	11.9	14,531	28.6	28.3	23.0	12.8	7.2	20,153	13.5	23,307
Confectioneries (traditional, cakes, etc.)	Baseline	16.9	24.0	26.7	17.6	14.7	13,549	6.8	14,531	8.5	19.4	29.8	22.1	20.3	21,843	6.3	23,307
	Interim	12.0	17.9	25.2	22.2	22.7	13,478	7.2	14,531	6.6	13.3	23.8	25.4	30.9	21,591	7.4	23,307

We summarized in table 4 the results of long-term reproducibility and variation of the food intake frequency for five years. It contains Spearman’s correlation coefficients, agreement of the categories (exact agreement and agreement allowing one category difference), mean scores of intake frequency, longitudinal difference, age effect, secular trend, and subjective change for 33 food items. Spearman’s correlation coefficients ranged from 0.27 and 0.55, and the median was 0.38 in males and 0.39 in females. Correlation was highest for intake of beef (0.45 for males and 0.55 for females), milk (0.55, 0.54) and margarine (0.46, 0.54) both in males and females. The lowest Spearman’s correlation coefficients were observed for fruit juice (0.27, 0.29) and Chinese cabbage (0.30, 0.30) in both males and females.

**Table 4.** Spearman’s correlation coefficients, agreement of the categories, mean scores, longitudinal difference, age effect, secular trend, and subjective change for 33 food items.

	Males									Females								
	
	SCC*	Agreement (%)		Mean score		Difference		Secular	Subjective	SCC*	Agreement (%)		Mean score		Difference		Secular	Subjective
						
		Exact	Allowing one category difference	Baseline	Interim	Longitudinal	Age effect^†^	trend	change		Exact	Allowing one category difference	Baseline	Interim	Longitudinal	Age effect^†^	trend	change
Beef	0.45	47.8	90.5	2.27	2.33	0.05	0.05	0.01	-0.14	0.55	51.3	91.3	2.27	2.28	0.02	0.02	0.00	-0.20
Pork	0.41	45.7	89.0	2.77	2.69	-0.08	-0.06	-0.02	-0.16	0.48	48.0	89.6	2.76	2.70	-0.06	-0.12	0.06	-0.22
Ham and sausages	0.41	40.8	84.4	2.43	2.38	-0.06	-0.06	0.00	-0.15	0.42	42.5	83.9	2.38	2.33	-0.05	-0.11	0.06	-0.24
Chicken	0.37	45.4	89.4	2.69	2.64	-0.06	0.00	-0.06	-0.08	0.39	47.5	89.7	2.79	2.71	-0.08	-0.03	-0.05	-0.11
Liver	0.40	56.9	91.9	1.77	1.76	-0.01	0.00	-0.02	-0.13	0.47	61.4	92.5	1.69	1.66	-0.02	0.00	-0.02	-0.17
Eggs	0.43	47.0	84.4	4.04	3.98	-0.06	0.00	-0.06	0.03	0.42	45.9	84.4	3.99	3.88	-0.11	-0.05	-0.06	-0.03
Milk	0.55	50.7	74.7	3.47	3.60	0.13	0.05	0.08	0.10	0.54	54.3	77.2	3.67	3.87	0.20	-0.01	0.21	0.16
Yogurt	0.38	57.9	81.5	1.57	1.79	0.22	0.05	0.17	-0.02	0.44	45.0	75.1	1.90	2.30	0.40	0.00	0.40	0.04
Cheese	0.44	53.1	87.3	1.72	1.82	0.10	-0.02	0.12	-0.09	0.49	55.1	86.8	1.71	1.86	0.15	-0.05	0.20	-0.11
Butter	0.38	51.9	83.9	1.78	1.77	-0.01	0.00	-0.02	-0.13	0.42	51.8	83.6	1.86	1.83	-0.03	-0.05	0.02	-0.18
Margarine	0.46	49.8	80.3	2.09	2.02	-0.07	0.01	-0.08	-0.10	0.54	46.6	78.4	2.48	2.35	-0.13	-0.07	-0.05	-0.13
Deep fried foods	0.35	45.5	88.9	2.94	2.95	0.00	-0.01	0.01	-0.08	0.36	46.1	89.7	2.90	2.88	-0.02	-0.04	0.02	-0.20
Fried vegetables	0.37	41.4	85.0	3.26	3.32	0.07	0.03	0.03	0.02	0.41	42.3	86.2	3.30	3.37	0.07	0.00	0.07	-0.04
Flesh fish	0.37	43.4	85.4	3.73	3.71	-0.03	0.01	-0.04	0.08	0.40	44.5	87.0	3.76	3.72	-0.04	-0.02	-0.03	0.09
Dried/salted fish	0.38	37.9	82.5	2.96	3.07	0.11	0.00	0.11	-0.05	0.39	37.8	81.8	2.90	3.02	0.12	-0.02	0.14	-0.08
Fish paste	0.39	41.3	85.2	2.36	2.32	-0.04	0.03	-0.07	-0.10	0.40	42.5	85.6	2.44	2.38	-0.06	-0.01	-0.05	-0.16
Green-leafy vegetables	0.32	38.8	81.5	3.81	3.77	-0.04	0.05	-0.09	0.10	0.34	42.6	84.4	3.98	3.93	-0.05	0.04	-0.09	0.13
Carrots and squash	0.35	37.8	82.3	3.17	3.26	0.09	0.03	0.06	0.06	0.36	40.8	85.8	3.59	3.63	0.04	0.00	0.04	0.12
Tomatoes	0.40	36.0	77.8	2.90	3.03	0.13	0.03	0.09	0.04	0.39	35.8	76.4	3.10	3.28	0.18	-0.02	0.20	0.06
Cabbage and lettuce	0.33	39.4	83.7	3.62	3.57	-0.05	0.00	-0.05	0.08	0.36	41.6	85.3	3.84	3.76	-0.09	-0.04	-0.05	0.08
Chinese cabbage	0.30	36.3	79.8	3.28	3.20	-0.08	0.04	-0.12	0.01	0.30	35.5	78.0	3.29	3.15	-0.14	0.03	-0.17	-0.02
Edible wild plants	0.33	38.4	80.4	1.97	2.34	0.37	0.00	0.37	-0.05	0.31	37.2	78.5	1.91	2.35	0.44	0.02	0.42	-0.07
Mushrooms	0.30	39.3	83.5	2.78	2.79	0.00	0.04	-0.03	0.02	0.35	39.5	83.8	3.05	3.03	-0.02	0.00	-0.02	0.06
Potatoes	0.39	39.4	84.1	3.08	3.15	0.07	0.05	0.02	0.01	0.38	42.3	86.2	3.46	3.43	-0.04	0.00	-0.04	0.03
Seaweeds	0.34	37.8	80.3	3.59	3.43	-0.17	0.05	-0.21	0.08	0.36	40.3	82.5	3.88	3.67	-0.21	-0.01	-0.20	0.11
Pickled vegetables	0.42	52.9	79.8	4.12	4.18	0.06	-0.01	0.07	-0.04	0.40	55.9	80.6	4.19	4.27	0.07	-0.02	0.09	-0.08
Tsukudani (food boiled with soy)	0.33	35.0	76.9	2.47	2.59	0.12	0.02	0.09	-0.10	0.37	36.1	77.1	2.39	2.54	0.16	0.03	0.13	-0.15
Boiled beans	0.37	40.3	82.9	2.34	2.46	0.12	0.11	0.02	-0.06	0.35	40.9	82.6	2.48	2.56	0.08	0.09	0.00	-0.08
Tofu (soybean curd)	0.42	44.5	87.4	3.76	3.84	0.07	0.03	0.04	0.13	0.43	47.3	88.6	3.91	3.95	0.04	-0.02	0.05	0.17
Oranges	0.40	34.7	75.9	3.33	3.26	-0.08	0.07	-0.15	0.01	0.37	38.3	76.5	3.88	3.67	-0.21	0.04	-0.25	0.02
Fruits other than oranges	0.37	36.8	77.3	3.48	3.42	-0.07	0.02	-0.09	0.05	0.36	41.5	79.0	3.96	3.85	-0.11	-0.03	-0.08	0.08
Fruit juice	0.27	29.9	67.5	2.90	2.37	-0.53	-0.02	-0.51	-0.05	0.29	29.5	64.4	2.97	2.42	-0.56	-0.04	-0.52	-0.09
Confectioneries (traditional, cakes, etc.)	0.46	34.4	73.6	2.89	3.26	0.37	0.09	0.28	-0.07	0.39	34.7	73.5	3.26	3.61	0.34	0.01	0.34	-0.14

* : Spearman’s correlation coefficient.

†: Age effect. Mean difference in five years at baseline.

Exact agreement varied 29.5-61.4% (median 40.8 in males and 42.5% in females), and was the highest for liver (56.9%, 61.4%) and pickled vegetables (52.9%, 55.9%) in males and females. Agreement allowing one category difference varied 64.4-92.5% with a median of 82.9% in males and 83.8% in females. It was also the highest for liver (91.9%, 92.5%) followed by beef (90.5%, 91.3%). On the other hand, exact agreement was lowest for juice (67.5%, 64.4%), followed by confectioneries (traditional, cakes, etc.) (73.6%, 73.5%).

**Figure 1.**
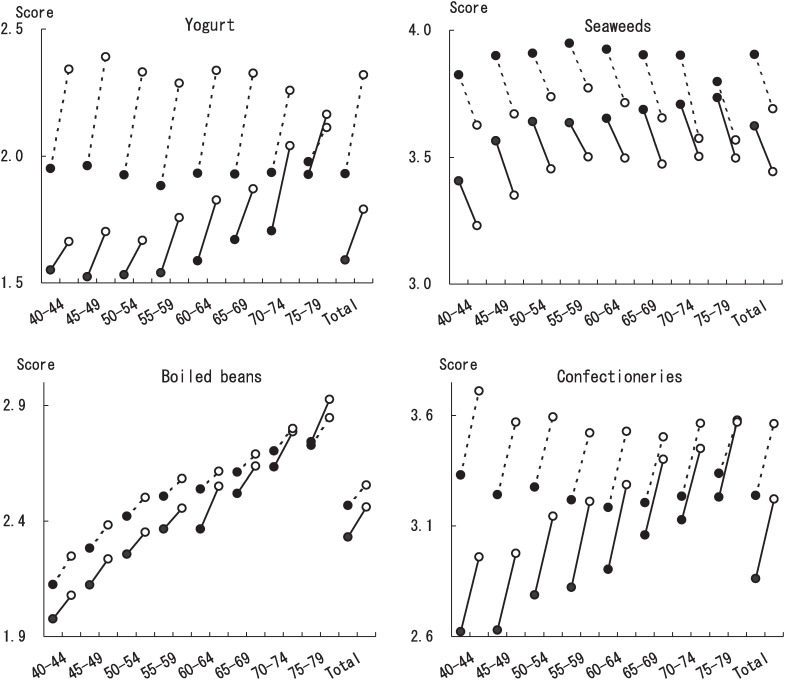
Intake changes over five years of yogurt, seaweeds, boiled beans and confectioneries in age groups. Closed circle and open circle stand for baseline and interim score, respectively. Solid line and dotted line stand for males and females, respectively.

Variations over five years which could not be assessed from Spearman’s correlation coefficients or agreement were evaluated using the difference of two scores (Table 4). The most increased food items were edible wild plants (0.37, 0.44) and confectioneries (0.37, 0.34). Intake of yogurt was increased in females (0.40) but to a lesser extent in males (0.22). Conversely, intake frequency was decreased most for fruit juice (-0.53, -0.56) followed by seaweeds (-0.17, -0.215). Figure 1 shows intake changes over five years for yogurt, seaweeds, boiled beans, and confectioneries in every five-year age group by sex.

The age effect was defined as the difference between the food intake frequency score and the mean score of the subjects who belong to one-rank older sub-cohort in this study. The age effect was the largest for boiled beans (0.11 in males, 0.09 in females), indicating that aged people consume boiled beans more often than younger people. This was followed by confectioneries (0.09), oranges (0.07) in males, by oranges (0.04) and spinach (0.04) in females. In the opposite direction, age effect was the strongest for pork (-0.06 in males, -0.12 in females) followed by ham and sausage (-0.06 in males, -0.11 in females). Younger subjects consume these items more often than aged subjects.

Secular trend was also most increased for edible wild plants both in males and females (0.37 in males, 0.42 in females) followed by confectioneries (0.28, 0.34), and yogurt in females (0.40). Here again it was decreased most for fruit juice (-0.51, -0.52) followed by seaweeds (-0.21, -0.20) and oranges (-0.15, -0.25). The results are almost identical to those for longitudinal differences.

The changes over five years were consistent between males and females. Spearman’s correlation coefficients of these indexes were high; Spearman’s correlation coefficient of the intake frequency at the baseline and interim questionnaire (0.79), exact agreement (0.86), agreement allowing one rank difference (0.91), longitudinal difference (0.91), age effect (0.81), secular trend (0.89), and subjective variation over five years (0.96) of 33 food items.

In spite of the consistent results between males and females, the subjective difference and longitudinal difference from the scores were poorly related. Spearman’s correlation coefficients were highest for milk (0.26 for males and 0.24 for females) and yogurt intake (0.20, 0.24), and lowest for pork (0.08, 0.09). Not only was there a poor correlation, but the direction of mean variation of longitudinal change and subjective change was inconsistent for 18 items for males and 17 for females among 33 items.

## DISCUSSION

In the present study, long-term reproducibility of food intake frequency after five years was assessed using Spearman’s correlation coefficients and agreement from basement and interim questionnaires. If both indexes are high, intake frequency is quite stable over five years. If only agreement is high, it could be due to a cluster of distribution. In this study, liver and pickled vegetable are clustered in the lowest (almost none) and highest (almost every day) category, respectively.

Spearman’s correlation coefficients in the study varied from 0.27 to 0.55, and agreement from 29.9% to 61.4%. Correlation was relatively higher for meat and dairy products and lower for vegetables and fruits in this study. The short-term reproducibility of the questionnaire in this study has been evaluated^11^. Compared to the correlation coefficients 0.57-0.94, and agreement 55%-80% of questionnaires with an interval of one week, the correlations in this study were lower. In Finland, intraclass correlation coefficient of 32 foods over 4-7 years varied 0.10-0.54 (median: 0.36), while that over 4-8 months varied 0.25-0.85.^7^ The median value is quite similar to our data. The decrease is considered to be due to a real change during the five years. Therefore, for valid evaluation for exposure, additional information on food intake would be needed over a long time course.

Overall increase or decrease which cannot be evaluated by correlation was assessed using a mean change in the score of intake frequency, and it was tested by the paired t-test. The longitudinal difference includes the effect of aging component, and we assumed the aging component could be substituted by the cross-sectional difference from one specific age sub-cohort to the one higher by one rank (five years). Thus the secular trend score was expressed by (longitudinal difference) - (age effect). For boiled beans, pork, ham and sausage intake, the age effect was larger than the secular trend. Especially, boiled bean intake was almost fully explained by the age effect. The trends were consistent between males and females.

The largest secular increase was observed for edible wild plants, yogurt and confectioneries in both males and females. Among them edible wild plants showed low correlation coefficients. Other than that, correlations were not so low, meaning that intake was increased as a whole while maintaining the relative order. On the other hand, fruit juice, orange and seaweed intake was considerably decreased. Decrease of orange intake frequency is consistent with the results from the national nutrition surveys^12^^,^^13^ in 1989 and 1994 (46.8g to 36.9g per day). However the intake of seaweeds is not greatly changed (5.9g to 5.8g). Intake of fruit juice is more inconsistent. It increased by 62% (6.6g to 10.7g) in the national surveys in this period. This discrepancy might be due to the difference in expression of the baseline and interim questionnaires. Only the baseline questionnaire included the comment of ‘in summer’ for fruit juice. It is consumed more in the hot season, and this comment caused the answer to be biased toward a larger score.^1^^,^^5^ Other than this item, seasonal effect did not distort the change in frequency, since they were asked average intakes in a past year, and the distributions of season of both surveys were not different so much (data not shown).

Subjective change of food intake frequency was poorly correlated to the longitudinal change of the same item. Subjectively increased items were tofu, spinach, milk, fresh fish, cabbage and lettuce, and seaweeds, while decreased items were pork, ham and sausage, beef, liver, and butter. These items are recommended to be consumed or avoided for healthy life, and responders’ desire for health might have distorted the real intake status. It also could be due to unclear wording of questions about dietary change. The questions did not specify whether the change was in frequency or amount. Confusion between frequency and amount could weaken the relationship. Or responder paid little attention to the time frame of five years and answered changes in terms of a shorter time frame.^14^ Whether the reason, the poor correlation generates serious misclassification if the subjective change is used in the regression analysis and might lead to biased results. To use data on change of dietary habit, information should be obtained twice and the difference evaluated.

In the interim survey, only 42.1% subjects of the baseline survey participated. However, the proportion from the 18 areas where all participants of the baseline survey were targeted to the interim survey was 78.8%, which can be interpreted as the response rate. Furthermore 81.1% subjects of the interim survey were from these areas. Thus, the problem of self-selection bias, which violates external validity, seems not to be serious in this study.

In conclusion, food intake was considerably changed over five years. Interim data should be considered for long-term follow-up study for a more valid evaluation of exposure. Subjective changes have a weak correlation to actual changes in food intake.

## MEMBER LIST OF THE JACC STUDY GROUP

The present investigators involved, with the co-authorship of this paper, in the JACC Study and their affiliations are as follows: Dr. Akiko Tamakoshi (present chairman of the study group), Nagoya University Graduate School of Medicine; Dr. Mitsuru Mori, Sapporo Medical University School of Medicine; Dr. Yutaka Motohashi, Akita University School of Medicine; Dr. Ichiro Tsuji, Tohoku University Graduate School of Medicine; Dr. Yosikazu Nakamura, Jichi Medical School; Dr. Hiroyasu Iso, Institute of Community Medicine, University of Tsukuba; Dr. Haruo Mikami, Chiba Cancer Center; Dr. Yutaka Inaba, Juntendo University School of Medicine; Dr. Yoshiharu Hoshiyama, University of Human Arts and Sciences; Dr. Hiroshi Suzuki, Niigata University School of Medicine; Dr. Hiroyuki Shimizu, Gifu University School of Medicine; Dr. Hideaki Toyoshima, Nagoya University Graduate School of Medicine; Dr. Kenji Wakai, Aichi Cancer Center Research Institute; Dr. Shinkan Tokudome, Nagoya City University Graduate School of Medical Sciences; Dr. Yoshinori Ito, Fujita Health University School of Health Sciences; Dr. Shuji Hashimoto, Fujita Health University School of Medicine; Dr. Shogo Kikuchi, Aichi Medical University School of Medicine; Dr. Akio Koizumi, Graduate School of Medicine and Faculty of Medicine, Kyoto University; Dr. Takashi Kawamura, Kyoto University Center for Student Health; Dr. Yoshiyuki Watanabe, Kyoto Prefectural University of Medicine Graduate School of Medical Science; Dr. Tsuneharu Miki, Graduate School of Medical Science, Kyoto Prefectural University of Medicine; Dr. Chigusa Date, Faculty of Human Environmental Sciences, Mukogawa Women’s University ; Dr. Kiyomi Sakata, Wakayama Medical University; Dr. Takayuki Nose, Tottori University Faculty of Medicine; Dr. Norihiko Hayakawa, Research Institute for Radiation Biology and Medicine, Hiroshima University; Dr. Takesumi Yoshimura, Fukuoka Institute of Health and Environmental Sciences; Dr. Akira Shibata, Kurume University School of Medicine; Dr. Naoyuki Okamoto, Kanagawa Cancer Center; Dr. Hideo Shio, Moriyama Municipal Hospital; Dr. Yoshiyuki Ohno, Asahi Rosai Hospital; Dr. Tomoyuki Kitagawa, Cancer Institute of the Japanese Foundation for Cancer Research; Dr. Toshio Kuroki, Gifu University; and Dr. Kazuo Tajima, Aichi Cancer Center Research Institute.
